# Attitudes of Company Executives toward a Comprehensive Workplace Health Management—Results of an Exploratory Cross-Sectional Study in Germany

**DOI:** 10.3390/ijerph182111475

**Published:** 2021-10-31

**Authors:** Achim Siegel, Aileen C. Hoge, Anna T. Ehmann, Peter Martus, Monika A. Rieger

**Affiliations:** 1 Institute of Occupational and Social Medicine and Health Services Research, University Hospital Tübingen, Wilhelmstr. 27, 72074 Tübingen, Germany; aileenhoge@msn.com (A.C.H.); anna.ehmann@med.uni-tuebingen.de (A.T.E.); monika.rieger@med.uni-tuebingen.de (M.A.R.); 2 Institute for Clinical Epidemiology and Applied Biometry, University Hospital Tübingen, Silcherstr. 5, 72076 Tübingen, Germany; peter.martus@med.uni-tuebingen.de

**Keywords:** workplace health management, total worker health, occupational safety and health, small and medium-sized enterprises (SME), cross-sectional survey, self-administered questionnaire, exploratory factor analysis, multiple regression analysis, Germany

## Abstract

Workplace health management (WHM) in Germany aims at maintaining and increasing the health and well-being of employees. Little is known about company executives’ attitudes toward WHM. To gain more insight, we conducted a large-scale survey in companies in the German county of Reutlingen in 2017. We sent a standardized questionnaire to 906 companies, containing inter alia 26 self-constructed declarative statements depicting company executives’ opinions on various WHM aspects; 222 questionnaires could be evaluated. By exploratory factor analysis we assigned the 26 items to six factors reflecting different attitudes toward WHM. Factor values were standardized to a scale from 0 to 10. The attitude ‘positive view of *general* health services in the company’, for example, achieved by far the lowest mean agreement (3.3 points). For the attitude ‘general skepticism toward WHM’, agreement and disagreement were balanced (5.0 points). Using multiple regression analyses, we searched for variables that could partially explain respondents’ agreement with attitudes. In conclusion, a general WHM skepticism was widespread, but not dominant. The idea that *general* health services should be offered in companies was predominantly rejected. Older respondents and respondents from smaller companies and craft enterprises were more skeptical than average about WHM and its possible extensions.

## 1. Introduction

### 1.1. Background

In Germany, workplace health management (in German: Betriebliches Gesundheitsmanagement) is often referred to as a guiding ideal for implementing a comprehensive combination of health-related measures in companies and other organizations. The term ‘workplace health management’ encompasses the integration and management of all operational processes in a company with the aim of creating healthy working conditions and promoting the health of its employees [1,2,3,4]. As this definition suggests, workplace health management is a holistic approach, and there is a strong similarity between workplace health management and the ‘Total Worker Health’ concept that has been promoted in the USA [5,6,7,8,9,10,11,12,13,14]. In recent years, this approach has further gained ground [15,16,17,18,19,20,21,22,23,24,25,26,27,28,29,30,31]. Workplace health management can be differentiated into the following four subcategories [4]: (1) occupational health and safety measures, (2) management of return-to-work processes of employees who were on long-term sickness absence (in short ‘reintegration management’), (3) workplace health promotion measures, and (4) supporting personnel development. In Germany, these subcategories differ as to legal status: many occupational health and safety measures and some reintegration measures are mandatory by German law and thus must be observed by companies; in contrast, measures in the other two areas—workplace health promotion and personnel development—are voluntary [4,32].

From numerous studies that have been conducted in Germany and other high-income countries in the preceding two decades we know that implementation of workplace health management measures depends on company size: the more employees a company has, the more likely it is that workplace health management measures are implemented [4,32,33,34,35,36,37,38,39,40,41,42]. In Germany’s small and medium-sized companies (for a definition cf. [43]) and in particular in small and micro enterprises, even legally required measures are often lacking [4,42,44,45]. Similar conditions seem to exist also in other industrialized high-income countries [46,47,48,49,50,51,52].

### 1.2. Different Attitudes in Society toward a Comprehensive Workplace Health Management?

The ideal of integrating traditional occupational health and safety with workplace health promotion and personnel development has been promoted for more than a decade by some scientists and political institutions alike, e.g., [48,53]. Concomitantly, arguments that “the workplace offers an ideal setting for health promotion” [54,55] and that “enterprises are an essential element of the national public health system” [53] have been put forward. In that spirit an editorial of *The Lancet Public Health* concluded in 2018: “The good news is that public health research into workplace-related health has now entered a new era. Establishing reliable evidence about what works to advance health, and perhaps more importantly what does not, offers the prospect for transformational change. The aim now will be to persuade employers that the health of their employees is an investment not a cost.” [54]

However, despite early attempts to identify facilitators, obstacles and barriers to implementing an integrative workplace health management (e.g., [33,56]), the results have been mixed (cf. above, Section 1.1)—even in Germany where the conception of workplace health management has a long tradition. As we reported above in detail, for Germany the bulk of small and micro enterprises still seem to stay at the sidelines. Thus the following question may be raised: is it possible that the condition of small businesses gives rise to and favors certain attitudes in managing directors which are adverse or at least disadvantageous to (some of) the fundamental ideas and implications of workplace health management? To give an example: the fundamental ideas of workplace health promotion and a comprehensive workplace health management may appear strange to those small business managers who think that health basically is—or should be—“a private matter for employees” [33]. To be sure, one might conceive still other—more down-to-earth—reasons for different attitudes among business leaders regarding workplace health promotion and management: while small and even many medium-sized companies often struggle to meet even legally required standards and regulations [4,41,42,44,45,57,58], large companies, which often have a professional in-house occupational health department, are in a much more favorable position: in most cases, it seems to be no problem for them to implement a comprehensive workplace health management and offer their own employees the entire range of standard occupational health management and health promotion measures. This means that in the competition for scarce, sought-after skilled workers, large companies—much more so than small and medium-sized enterprises—can benefit from the fact that, e.g., workplace health promotion offerings increase employee loyalty and motivation, even if such measures may not demonstrably promote employees’ health [59]. Such competitive disadvantages of small companies might as well contribute to a dubious image of workplace health promotion (and an integrative workplace health management) in small business leaders. A statement like, e.g., “workplace health promotion serves the image of the company rather than the health of the employees” could be a reflection of such an image and could well contribute to a negative or skeptical attitude toward workplace health management.

To sum up the preceding arguments, (1) it seems probable or at least possible that there is a real ’attitude divergence’ in society and in particular among business leaders regarding workplace health promotion and workplace health management, and (2) at the same time company executives’ attitudes might be a key to explaining why an integrative workplace health management has met a skeptical reaction among executives of smaller enterprises.

### 1.3. Objectives of the Study

Nonetheless, little is known from quantitative studies about managing directors’ and other executives’ attitudes toward workplace health management or toward an extension of the standard repertoire of health management measures. In two quantitative studies conducted more than a decade ago, German company executives were asked about specific motives and reasons for implementing or refraining from certain workplace health management measures [33,56]. Both studies, however, seem to be of limited use if one wants to know more about company executives’ attitudes that underlie their arguments in favor of or against certain measures; moreover, the more recent study [33] included only medium-sized enterprises, not considering smaller companies at all—although more than 40% of the German workforce is employed by small or micro enterprises [60]. In order to obtain recent information about company executives’ attitudes on various aspects of workplace health management, encompassing not only medium-sized or big companies, but also smaller ones, the authors conducted a survey in companies in the county of Reutlingen (Landkreis Reutlingen) in southwestern Germany in 2017. The county of Reutlingen is characterized by many economically strong medium-sized and some large companies, but also by more than 1000 small and more than 11,000 micro enterprises [61]. As to socio-economic strength and population health, the county of Reutlingen is above the German average: in the year of the survey the unemployment rate in the county was 3.5% (Germany: 5.7%), the average monthly household income amounted to 2044 € (Germany: 1882 €), while life expectancy at birth amounted to 82.27 years (Germany: 80.82 years) [62]. At the end of 2017, the county had 285,754 inhabitants [63].

Based on such a survey we wanted to answer the following research questions exploratively:

(1) What attitudes do company executives have toward comprehensive workplace health management?
(2) How common are those attitudes? In other words: how high is the level of company executives’ agreement with those attitudes?
(3) Which variables statistically explain company executives’ agreement with a given attitude?

While research question (1) is the foundation for answering the remaining two questions, the answers to research questions (2) and(3) are of high practical relevance: if, e.g., politicians or scientific advisors want to contribute to a change in a certain attitude among company executives—e.g., of a fundamental skepticism toward some basic ideas of workplace health management—it is important to know (a) how common this attitude is and (b) which factors explain the target group’s agreement with that attitude. In other words: while research questions (2) and (3) are logically subordinate to research question (1), they are of high practical relevance for designing a more effective policy in dealing with the acceptance of workplace health management among business executives.

## 2. Methods

### 2.1. Study Participants and Data Collection

In this survey we pursued an exploratory cross-sectional design. For the recruitment of study participants, we cooperated with two organizations: On the one hand, the Reutlingen Chamber of Crafts was willing to support us in recruiting the county’s craft enterprises (which are obligatory members of their regional chamber of crafts). The Reutlingen Chamber provided us with a dataset containing 277 addresses of all of the Chamber’s member enterprises with 10 or more employees. To recruit non-craft enterprises, on the other hand, we cooperated with a marketing agency and debt-collection company [64]. From this company we bought 632 addresses of the county’s non-craft enterprises with 20 or more employees. Thus altogether 909 addresses of potential study participants were available at the beginning of the study. This number included neither the county’s micro enterprises (fewer than 10 employees) nor the very small non-craft enterprises (fewer than 20 employees) as we supposed beforehand that most elements of workplace health management were not implemented in such small companies in any case. Nonetheless, those 909 enterprises comprised the large majority of all non-micro enterprises in the county: data from the Statistical Office of the German federal state Baden-Württemberg show that 1303 non-micro enterprises were registered there at the end of 2017 [61]. (At the same time, 11,406 micro enterprises were registered in the county [61]). As it turned out later, three of those 909 enterprises had ceased to exist at the beginning of study as three letters with invitations to participate in the study turned out to be undeliverable. Thus, the effectively invited study population consisted of 906 enterprises which means that about 70% of all non-micro enterprises in the county were invited.

In July and early August 2017, those 906 enterprises received a standardized questionnaire and an invitation letter which contained all relevant information on the study. Furthermore, we asked the recipient to hand out the questionnaire to either the managing director or an executive of the personnel department.

Non-craft enterprises received their questionnaire and study information directly from our institute, craft enterprises together with an additional motivating letter from the Reutlingen Chamber of Crafts (which supported the survey). Two weeks after the first invitation letter we sent a reminder to all of the potential responders, regardless of whether some of the enterprises had already filled in and returned their questionnaire.

According to the competent ethical committee at the University Hospital Tübingen, no formal ethical approval of the study was required (information provided by the Ethics Committee at the University Hospital of Tübingen on 25 November 2014—project number 697/2014VF). The potential respondents were informed that study participation was voluntary and that all of the surveyed data would be analyzed and published anonymously.

### 2.2. Standardized Questionnaire

The questionnaire was developed by a multidisciplinary team including a specialist in occupational medicine (MAR), a sociologist and public health researcher (AS), and a medical student (ACH). It contained standardized questions on (a) the implementation of various health-related measures in the company (including all types of workplace health management measures from the four subcategories mentioned above), (b) satisfaction with the implementation of these measures, (c) respondents’ opinion on various aspects of workplace health management and related measures, and (d) respondents’ opinion on how to finance certain preventive measures; the questionnaire finally concluded with (e) information on the company and the person who filled in the questionnaire: here, sociodemographic data of the respondent and standardized information on company characteristics were gathered (such as, e.g., branch of industry, current number of employees, availability of an occupational health physician). Altogether, the questionnaire contained 121 items [65].

As the focus of this paper is on company executives’ attitudes toward a comprehensive workplace health management, parts (c) and (e) are of primary interest in the following. More precise descriptions of the questionnaire’s parts (a) and (b) as well as analyses focusing on these parts have been published elsewhere [4,58,65].

Part (c) of the questionnaire contains 26 declarative statements (items) on various aspects of workplace health management, ranging from basic statements underlying—or contradicting—the ‘philosophy’ of workplace health management (e.g., “Health is a private matter to each employee.”) to statements on specific preventive health offerings in one’s company (e.g., “All employees should be offered vaccinations for private travel in the company.”). These statements were developed by three authors of this paper (ACH, AS, MAR) in the course of several brainstorming and discussion meetings; the statements were to reflect a broad array of opinions and statements on workplace health management possibly shared by executives of small, medium-sized and big enterprises. Some opinions and statements were inspired by relevant literature [33,56]: thus, e.g., the statements “health is a private matter for each employee” or “it is sufficient for companies to fulfill the legal requirements for occupational health and safety and company reintegration management” referred to an earlier survey [33]. Some other opinions and statements, primarily statements contradicting or supporting the idea that the workplace should be perceived and used as an ideal setting for health promotion, were based on the senior author’s experiences from discussions with representatives of the Employers’ Association of the Metal and Electric Industry Baden-Württemberg (Südwestmetall). Still other items, such as “workplace health management serves the image of the company rather than the health of the employees” and “before society demands more commitment from companies, general health care should be improved”, were developed in the course of discussions between the three above-mentioned authors by sharpening arguments that can occasionally be heard by business leaders when discussing possible extensions of workplace health management measures. The full list of the 26 declarative statements is presented in Table 4 below.

The answers to these 26 declarative statements could be given in the form of a Likert-type scale, containing four levels of agreement (“fully agree”, “tend to agree”, “tend to disagree”, “fully disagree”) and one additional answer “cannot judge” (which was treated as a missing value).

Before the start of the study, the questionnaire was subjected to a pretest: a total of 24 participants in a further training event for personnel executives from the metal and electrical industry in southwestern Germany completed the preliminary questionnaire. Afterwards, they discussed possible ambiguities and other potential weaknesses of the questionnaire with one of the authors (AS). For further details of the pretest cf. [65].

### 2.3. Statistical Analysis

All statistical analyses were performed with SPSS, version 24 and 25 (IBM Analytics, IBM Corporation, Armonk, NY, USA).

#### 2.3.1. Exploratory Factor Analysis

We used principal component analysis with subsequent exploratory factor analysis (Varimax rotation) to reduce the above-mentioned 26 declarative statements to a lower number of underlying factors [66]. These factors should be interpretable as latent variables, in our context as ‘opinion patterns’ or ‘attitudes’ to workplace health management.

To assess suitability of those 26 items for exploratory factor analysis, we used the Kaiser-Meyer-Olkin criterion (KMO): a KMO value of 0.6 or higher confirms sufficiently high correlations among the items and indicates the suitability of the data [66].

We performed the exploratory factor analysis with varimax rotation in order to obtain a simple structure and to be able to assign the items to the factors as clearly as possible. Factor extraction was performed according to the Kaiser criterion, postulating an initial eigenvalue > 1 for a factor to be extracted. (This means that we extracted only factors which explained more total variance than a single standardized variable.) The level of the factor loadings determines the assignment to the factors; in our case, the loadings had to be at least 0.4 [67,68].

#### 2.3.2. Construction of Scores Expressing the Degree of Agreement with Attitudes

The factors that resulted from the exploratory factor analysis should represent ‘opinion patterns’ or ‘attitudes’ of company executives towards workplace health management. To determine respondents’ degree of agreement with such attitudes, we computed agreement scores for each attitude (i.e., for each ‘factor’ consisting of several items). In case where a single declarative statement was formulated in a way that was opposite in content to the other statements of that factor (and thus loaded negatively on that factor), the response scale of this statement was reversed for the calculation of the score.

Accordingly, for a score variable to be formed for a given case (respondent), the respondent had to have given valid answers for at least three quarters of the items that constituted a given attitude. (Example: if a score variable consisted of four items, valid values had to be present for at least three items for a valid score value to be computed in that case. In such a case we computed the score value by multiplying the mean of those three items by the number of all items—here: four items—of the respective score.) Finally, we standardized all score variables so that every score variable had a theoretical value range of ‘0′ to ‘10′, with ‘10′ representing maximal agreement (“fully agree”) and ‘0′ representing minimal agreement (“fully disagree”) with a given attitude. Thus we followed the usual rules of constructing scores based on Likert-type scales [69].

#### 2.3.3. Multiple Regression Analyses to Determine Predictors of Respondents’ Agreement with Attitudes

To determine independent variables (potential predictors) for respondents’ agreement with a given attitude, we performed for each agreement score—i.e., each dependent variable—a multiple linear regression analysis using the method of backward elimination of independent variables (criterion: probability of F-value for elimination ≥0.050). For all final regression models, F values (and the respective *p* values), and corrected R^2^ values were determined for the regression models, while standardized regression coefficients (ß values) and their respective *p* values were determined for the statistically significant predictors. Following Cohen [70], we interpreted determination coefficients from 0.02 to 0.12 as indicative of a small amount of explained variance, from 0.13 to 0.25 as indicative of a moderate amount and from 0.26 as indicative of a high amount of explained variance. Corresponding to Cohen’s thumb rule for the interpretation of Pearson’s correlation coefficients, standardized regression coefficients (ß values) amounting from 0.10 to 0.29 were regarded as indicating a small effect, from 0.30 to 0.49 a moderate effect, and from 0.50 a large effect.

In all of these multiple regression analyses we considered the following variables as potential predictor variables:

(a) company size, i.e., current number of employees (continuous variable),
(b) type of business: (i) non-craft business vs. (ii) craft business (binary variable),
(c) type of industry branch [65]: (i) branch without increased work-related health hazards such as risk of occupational diseases (into this category we grouped energy and water supply, trade and commerce, hotels and restaurants, transport and communications, banking and insurance, real estate and housing, public administration, education and training, services and utilities) vs. (ii) branch with increased health hazards (into this category we grouped agriculture and forestry, fishing and fish farming, mining/stone/earth industry, production and manufacturing, construction, maintenance and repair, health and social services) (binary variable),
(d) availability of an occupational health physician /company doctor (binary variable),
(e) respondent’s position within the company: (i) managing director vs. (ii) personal department member vs. (iii) other position (categorical variable; transformed into three dummy variables for the regression analyses),
(f) age of respondent (continuous variable), and
(g) gender of respondent (binary variable).

A short note seems to be appropriate regarding (c): the categorization of industry branches into (i) or (ii) was based on an expert rating by one of the authors (MAR, who is a specialist in occupational medicine). The rating answered the question of whether a given industry or branch was required to have mandatory preventive employee screening according to the German Ordinance on Preventive Occupation Health Care [71]—a question which is in itself related to the fact that some industries have an increased risk of work-related illnesses and occupational diseases.

## 3. Results

### 3.1. Participants and Sample Characteristics

The overall response rate in the study was 24.5% (222/906). As can be seen from Table 1, the response rate differed according to company size (i.e., number of employees). The majority of study participants (57.2%; 127/222) represented small enterprises with up to 50 employees. Just under half of the companies represented in the survey (48.2%; 107/222) had an occupational health physician (either employed or commissioned) at the time of the survey, but more than three-quarters of the companies indicated that they had an occupational safety engineer (76.1%; 169/222).

As for industry affiliation, nearly one third of participating companies belonged to the production or manufacturing branch (n = 67; 30.2%) and 37 companies (16.7%) to the construction industry. Another 34 participating companies (15.3%) and 31 companies (14.0%) represented services and trade, respectively. The remaining 53 (23.9%) participants belonged to various other branches (for more details cf. [4], p. 6).

The socio-demographic characteristics of those persons who filled the questionnaire for a given company are shown in Table 2.

### 3.2. Results of the Exploratory Factor Analysis: Attitudes toward Workplace Health Management

This Section provides an answer to the first research question (cf. above, Section 1.3).

From the exploratory factor analysis, a KMO value of 0.85 resulted which demonstrated the suitability of our data for an exploratory factor analysis and for extracting a reduced number of underlying factors from those 26 items. This result was confirmed by the result of the Bartlett test (*p* < 0.001) indicating that the correlations between the items were sufficiently large.

Using exploratory factor analysis, six factors with an eigenvalue above 1 (Kaiser criterion) were extracted. These six factors explained a total variance of 61.4%; details are displayed in Table 3.

In Table 4 we present in detail which individual items (declarative statements) were related to which of the six factors. Note that items appear in Table 4 in an order which corresponds (a) to their affiliation to factors 1 to 6 and (b) to their respective factor load (which is not the order in which they appeared in the questionnaire). Table 4 shows that all items had a factor load of >0.4 and hence could be attributed to one factor.

Only 2 out of 26 items loaded similarly high on more than one factor, indicating that they could have been attributed to more than one factor. This concerns item no. 2.7 (cf. Table 4) which was attributed to factor 2 but loaded similarly high on factor 3 (−0.43 on factor 2 and 0.41 on factor 3) and item no. 5.3 that was attributed to factor 5 but loaded similarly high on factor 4 (0.451 on factor 5 and 0.447 on factor 4).

The six factors could be interpreted fairly coherently as six different overarching attitudes toward workplace health management and were named accordingly (cf. Table 4, left column, and Table 5): factor 1 was called ‘positive view on *general* health services in the company’, factor 2 ‘workplace health management and workplace health promotion create added value to companies’, factor 3 ‘general skepticism toward workplace health management’, factor 4 ‘positive view on companies as setting for health promotion’, factor 5 ‘positive view on occupational safety and health according to corporate social responsibility’, and factor 6 ‘positive view on carrying out minor medical checks at the company doctor’s practice’.

### 3.3. Respondents’ Agreement with Attitudes toward Workplace Health Management

This Section provides the answer to the second research question (cf. above, Section 1.3).

After the extraction and interpretation of the six factors we computed scores that expressed study participants’ degree of agreement with each of these six factors (attitudes). The results are shown in Table 5 (for methodological details cf. Section 2.3.2).

As Table 5 shows, agreement with the attitude ‘positive view on occupational safety and health according to corporate social responsibility’ was highest on average at 7.86 points; moreover, the low standard deviation of 1.48 points illustrates a low level of divergence among respondents—i.e., the high level of agreement is relatively unanimous. In contrast, agreement with the attitude ‘positive view of *general* health services in the company’ was lowest on average at 3.26 points. As to the other four attitudes, agreement and disagreement roughly balanced each other out: here, the average agreement ranged between 4.72 and 6.08 points, with attitude ‘workplace health management and workplace health promotion create added value to companies’ tending to the positive pole (6.08 points on average).

### 3.4. Predictors of Respondents’ Agreement with Attitudes toward Workplace Health Management

This Section provides the answer to the third research question (cf. above, Section 1.3).

Multiple linear regression analyses were conducted using the backward elimination method—as set out in detail in Section 2.3.3—to check which predictor variables statistically explained respondents’ agreement with the six attitudes.

For all six attitudes (dependent variables) we checked all seven independent variables mentioned above in Section 2.3.3. For five attitudes (attitudes 1 to 3, 5, and 6—cf. Table 4) statistically significant regression models with several predictor variables could be detected. Only for respondents’ agreement with attitude 4 (‘positive view on companies as a setting for health promotion’) were no statistically significant predictors found. In the following, we summarize the results for each dependent variable.

#### 3.4.1. Predictors of Respondents’ Agreement with Attitude ‘Positive View on *General* Health Services in the Company’

For the dependent variable ‘positive view on *general* health services in the company’ we found a statistically significant regression model with a small amount of explained variance: F (2, 174) = 8.273, *p* < 0.001; corrected R^2^ = 0.08. Two statistically significant predictors were found: respondents’ age (ß = −0.23, *p* = 0.002) had a small negative effect on the dependent variable (i.e., the older the respondents, the less they agreed with that attitude), whereas company size had a small positive effect (ß = 0.19, *p* = 0.010) on the dependent variable (i.e., the more employees the company had, the more the respondents agreed with that attitude).

#### 3.4.2. Predictors of Respondents’ Agreement with Attitude ‘Workplace Health Management and Workplace Health Promotion Create Added Value to Companies’

As to respondents’ agreement with the attitude ‘workplace health management and workplace health promotion create added value to companies’, a statistically significant regression model with a high amount of explained variance resulted: F (3, 158) = 20.301, *p* < 0.001; corrected R^2^ = 0.27. Three statistically significant predictors were found: company size had a moderate positive effect (ß = 0.36, *p* < 0.001) on the dependent variable, respondent’s age a small negative effect (ß = −0.26, *p* < 0.001), and type of business a small negative effect (ß = −0.27, *p* < 0.001), too (i.e., respondents from craft businesses agreed less with that attitude than respondents from non-craft businesses).

#### 3.4.3. Predictors of Respondents’ Agreement with Attitude ‘General Skepticism toward Workplace Health Management’

For respondents’ agreement with the attitude ‘general skepticism toward workplace health management’, we found a statistically significant regression model with a small amount of explained variance: F (3, 171) = 7.106, *p* < 0.001; corrected R^2^ = 0.10. There were three statistically significant predictors: company size had a small negative effect (ß = −0.17, *p* = 0.020), type of business a small positive effect (ß = 0.16, *p* = 0.034; i.e., respondents from craft businesses had a more skeptical attitude toward workplace health management than respondents from non-craft businesses), whereas the availability of an occupational health physician had a small negative effect (ß = −0.16, *p* = 0.036; i.e., skepticism was reduced when an occupational health physician was available).

#### 3.4.4. Predictors of Respondents’ Agreement with Attitude ‘Positive View on Occupational Safety and Health according to Corporate Social Responsibility’

As to respondents’ agreement with a ‘positive view on occupational safety and health according to corporate social responsibility’, a statistically significant regression model with a small amount of explained variance resulted: F (2, 166) = 6.044, *p* = 0.003; corrected R^2^ = 0.06. Two statistically significant predictors were found: company size had a small positive effect (ß = 0.20, *p* = 0.011), whereas type of business had a small negative effect (ß = −0.16, *p* = 0.040).

#### 3.4.5. Predictors of Respondents’ Agreement with ‘Positive View on Carrying Out Minor Medical Checks at the Company Doctor’s Practice’

For respondents’ agreement with the attitude ‘positive view on carrying out minor medical checks at the company doctor’s practice’, we found a statistically significant regression model with a small amount of explained variance: F (2, 148) = 6.999, *p* = 0.001; corrected R^2^ = 0.07. There were two statistically significant predictors: If respondents worked in the personnel department of the company, this had a small positive effect (ß = 0.26, *p* = 0.001), while company size had a small negative effect (ß = −0.17, *p* = 0.034).

## 4. Discussion

### 4.1. General Aspects

Altogether 222 (out of 906 effectively invited) company representatives took part in this questionnaire-based study. This response rate (24.5%) might seem moderate or even low to moderate, but it is well within the range which is common for postal surveys that do not provide special incentives for participation (e.g., [73,74,75,76]). Most respondents of the study (57%) represented small companies with a maximum of 50 employees—thus, we do not have a pronounced ‘medium-sized company’ or even ‘large company bias’ in our sample despite the fact that we did not survey micro enterprises with fewer than 10 employees (cf. Section 2.1 above).

From 26 declarative statements (questionnaire items) with which respondents expressed their opinions on various aspects of workplace health management (and its possible extensions), we extracted—using an exploratory factor analysis—six underlying factors. These factors could be interpreted as attitudes toward (certain aspects of) workplace health management. The statistical details of this exploratory factor analysis (e.g., a KMO value of 0.85 and an explained variance of 61.4% by the six factors) and the contextual plausibility of the six factors show that it is possible and reasonable to derive such attitudes from a multitude of declarative statements. An important methodological advantage of assigning a multitude of declarative statements (or opinions) to a smaller number of underlying attitudes is that scores expressing agreement with given attitudes are less prone to bias than just median or mean values of individual declarative statements.

### 4.2. Remarks on Individual Attitudes, Respondents’ Agreement with Attitudes, and the Predictors Explaining Respondents’ Agreement with Attitudes

Thus, e.g., regarding the attitude ‘positive attitude toward occupational health and safety according to corporate social responsibility’ includes three different declarative statements; these three statements can be considered as ‘commonplaces’ that virtually every company representative should be able to agree with. Consequently, the mean agreement with this attitude was the highest (7.86 points on a scale between 0 and 10) of all the attitudes presented here, and the low standard deviation (cf. Table 5 above) demonstrates that this agreement was comparably unanimous. Thus, it is hardly surprising that our regression analyses, with which we searched for variables that (partially) explained agreement with that attitude, resulted in a model with a very small amount of explained variance (corrected R^2^ = 0.06). According to the model, agreement with this attitude can be explained to a small extent by company size (small positive effect) and type of business (small negative effect, i.e., respondents from craft businesses agreed less with that attitude).

In contrast—and not surprising—the attitude ‘positive view on *general* health services in the company’, composed of six declarative statements, reached the lowest agreement mean (3.26 points). As the majority of study participants represented small enterprises and as many small enterprises in Germany struggle to meet even basic occupational health and safety standards which are required by law [4,42,44,45], it is well understandable that this attitude was rather not agreed with in our sample: for small companies and many medium-sized enterprises, too, an implementation of general health services in the company would mean a disproportionately high effort. Our regression analyses, with which we searched for variables that could explain the agreement with that attitude, showed that agreement with this attitude could be explained to a small extent by respondents’ age (small negative effect) and company size (small positive effect).

The attitude ‘workplace health management and workplace health promotion create added value to companies’ consists of seven declarative statements. The mean agreement score of this attitude was 6.08, i.e., the mean agreement clearly tends to the positive pole of the scale. As to the predictor variables regarding this attitude, company size had a moderate positive effect, respondents’ age a small negative effect, and type of business (non-craft vs. craft business) also a small negative effect.

The attitude ‘general skepticism toward workplace health management’, embodied by four items, had a mean agreement score of 5.02, i.e., it was right in the middle of the theoretical range. While company size and the availability of an occupational health physician had a small negative effect on skepticism, type of business (non-craft vs. craft business) had a small positive effect. It should be noted that the individual items that make up this attitude (cf. Table 4, Section 3.2) do not seem to be perfectly homogeneous: whereas the first three items do express a fundamental criticism of workplace health management —“workplace health management serves the image of the company rather than the health of the employees”, “health is a private matter for each employee” and “before society demands more commitment from companies, general health care should be improved” –, the fourth item expresses a rather pragmatic and thus ‘non-fundamentalist’ critique (“workplace health management is difficult to implement in company practice”) that has been regularly voiced in earlier company surveys and in a recent qualitative study [33,56,77].

The remaining two attitudes—‘positive view on company as a setting for health promotion’ (four items) and ‘positive view on carrying out minor medical checks at the company doctor’s practice’ (two items)—reached moderate mean agreement scores of 4.72 and 4.92, respectively. For respondents’ agreement with a ‘positive view on carrying out minor medical checks at the company doctor’s practice’ we found two predictors that explained agreement with that attitude to a small extent: while respondents’ position in the company as a personnel department member had a small positive effect, company size in itself had a small negative effect. As to ‘positive view on company as a setting for health promotion’ we did not detect any statistically significant independent variables. Remarkably, the enthusiasm of many scientists about companies as an ideal setting for health promotion ([48,53,54,55], cf. above, Section 1.2) is not generally shared by the respondents of this study: the mean agreement with the concerning attitude (‘positive view on company as setting for health promotion’) amounts to only 4.72, which may be interpreted as neutral with a slight tendency to the negative pole. Interestingly, company size in this case does not significantly predict a difference in agreement—contrary to most other attitudes. This means that respondents’ hesitancy toward that attitude is by no means limited to small business leaders.

Thus, for five out of six attitudes, we did detect statistically significant predictor variables. Company size was detected four times as a significant predictor: all in all (with the exception of attitude ‘positive view on carrying out minor medical checks at the company doctor’s practice’), it had the effect that the larger the company, the more positive were the company representatives’ attitudes toward certain aspects of workplace health management and its possible extensions. These results seem plausible and reasonably in line with other studies on the subject in that company size has often been demonstrated as a variable that statistically explained the grade of implementation of workplace health management measures [4,7,32,33,34,35,36,37,38,39,40,41,42,47,51,78].

In contrast to company size, the predictors ‘type of business’ (non-craft vs. craft business) and ‘age of respondent’ each had an effect in the opposite direction in three or two cases, respectively: if respondents represented a craft business (and the older the respondents), the more skeptical the respondents were toward certain aspects of comprehensive workplace health management.

To the best of our knowledge, the age of company executives has been identified in only one earlier study as a variable that is significantly associated with a lower degree of implementation of workplace health management measures [56]. The author of that study summarized this point as follows: “Younger managers (<40 years of age) tend to be more aware of the need for workplace health promotion and pursue it both more intensively and more regularly than their older colleagues. On average, younger managing directors implemented organizational and personnel development measures around three times more frequently than older managing directors.” ([56] p. 23, translation by A.S.). At this point we should, however, resist the temptation to conclude from such results that the relationship between company executives’ age and that kind of skepticism is only a ‘generation effect’, concluding that a new generation of executives inevitably makes this relationship disappear. That relationship might reflect, at least to some degree, an ‘age effect’ in that growing old—and at the same time, gathering more and manifold experiences in life—leads older company executives to an, e.g., more pronounced skepticism (or to less idealism) toward projects or measures that are proposed or prescribed by well-intentioned advisors or sociopolitical stakeholders. In any case, we recommend a more profound exploration of this relationship in future studies on that topic.

As far as we know, type of business (non-craft vs. craft business) has so far not been identified in a multivariate analysis as a significant predictor explaining skepticism toward workplace health management A sound and convincing interpretation of this result deserves a deeper analysis than we are able to deliver at this moment. An explanation would probably have to consider the cultural-sociological dimension of the difference between modern industry, characterized by mass production and an increasingly flexible work, workforce and company management on the one hand [79], and the more traditional culture of craft work and craft enterprises on the other hand [80].

All in all, the predictors found in our regression analyses explained only in one case a substantial proportion of the dependent variable’s variance (‘workplace health management and workplace health promotion create added value to companies’, corrected R^2^ = 0.27); in the remaining four cases the corrected R^2^ values were very low, ranging between 0.06 and 0.10. Regarding these latter results—as well as the attitude for which we did not find any significant predictors—we must concede that either we did not measure all (or the most relevant) predictor variables for the concerning attitudes or those attitudes’ variance is largely determined by chance—which, certainly, can be doubted.

### 4.3. Contextual Aspects

This study was conducted in the county of Reutlingen in southwestern Germany, which is an economically powerful region (cf. Section 1.3 above). The region’s health authorities put a strong emphasis on workplace health management and support of companies—thus, e.g., regional health authorities offer health counselling and certification, particularly to small and medium-sized companies in the region [81]. Against this background, the widespread general skepticism toward workplace health management and the low positive attitude toward *general* health services in companies which we found in this study might suggest that this kind of skepticism is probably even more widespread in other regions with less favorable conditions.

### 4.4. Strengths of the Study

Our sample covers almost all kinds of businesses and all economic branches including craft businesses of one German county. Likewise, this sample contains companies of all sizes apart from very small ones (i.e., fewer than 10 or 20 employees respectively—cf. Section 2.1). For that reason we think that the data of our sample show a more realistic picture of company representatives’ attitudes than usual surveys (which often focus on larger companies) or than pictures of glossy ceremonies that are dominated by company representatives applying for awards or financial support because of their ‘outstanding’ workplace health management (cf. e.g., the companies which have been awarded the “Corporate Health Award” [82] which is one of the most significant topic-related prizes for companies in Germany).

### 4.5. Limitations of the Study

As we used only cross-sectional data in our analyses, one should refrain from interpreting the findings of our regression analyses in terms of confirmed causal relationships. Consequently, one should not interpret the predictors of our regression models as ‘modifiable causal factors’.

It could seem at a first glance that the moderate (or low to moderate) response rate of our study—24.5%—limits the generalizability of our findings. This is certainly true in part. It is quite probable that our 24.5% sample represents a kind of ‘positive selection’ with regard to study participants’ attitudes toward workplace health management and its different components, in particular when we keep in mind that another main topic of the questionnaire was the grade of implementation of health-related measures in the company—including measures that were required by law [4,58]. Against that background it seems very plausible that this 24.5% sample represents rather a ‘positive’ selection of companies in the above-mentioned sense [4]. This would mean that the ‘real-world skepticism’ of company representatives toward a comprehensive workplace health management is probably even underestimated by our data—due to a probable selection bias. If this were the case, our main finding—that there is a substantial level of skepticism toward workplace health management among ordinary company representatives—would be even more clearly underlined.

Approximately the same effect—a ‘positive selection’ of companies in the sense mentioned in the preceding paragraph—is likely to have resulted from the study’s exclusion criteria: as detailed in Section 2.1, we excluded micro businesses (fewer than 10 employees) in both the craft and non-craft sectors and also excluded very small non-craft businesses (fewer than 20 employees). Since the results of this study show that company size is a predictor of company representatives’ approval of or skepticism towards comprehensive workplace health management (cf. Section 3.4), it seems quite likely that company representatives’ skepticism toward workplace health management and its possible extensions (cf. Table 5) would have been higher in this study if we had aimed for a sample representative of the county’s entire company population.

Another type of bias—a self-selection bias—could theoretically have also biased the results ‘into the positive.’ This self-selection bias could be conceived as follows: within a company invited to participate in the survey, the completion of the questionnaire could have been preferably delegated to (or voluntarily undertaken by) those persons with an above-average commitment to workplace health management and consequently with a rather positive attitude towards workplace health management. Though such a self-selection bias cannot be ruled out definitely, a self-selection bias connected with specific positions within a company (i.e., managing director vs. member of the personnel department vs. other position) seems rather unlikely since in our multivariate regression analyses respondents’ position within the company was only once revealed as a significant predictor for company representatives’ agreement with the six attitudes (cf. Section 2.3.3 and Section 3.4).

Finally, one general reservation as to the generalizability of the results of our exploratory factor analysis should be made: as the sample was recruited in just one German county (in which there are, as one could argue, special—rather favorable—conditions for workplace health management, cf. above), we should be cautious as to the generalizability and replicability of the results of our exploratory factor analysis. It cannot be excluded that an exploratory factor analysis based on those 26 items leads to other results if a similar survey is conducted in other German regions. We therefore recommend that those six factors which were extracted in our exploratory factor analyses should be verified in an independent sample.

## 5. Conclusions

To the best of our knowledge, this is the first study to examine the attitudes of managers of almost all company sizes toward comprehensive workplace health management on the basis of an exploratory factor analysis. Using the example of a region in southwestern Germany, the study investigated (1) the attitudes underlying a large number of individual opinions on occupational health management, (2) the extent to which managers agree with these attitudes, and (3) the variables that explain managers’ agreement with the individual attitudes.

The agreement scores as to the six attitudes toward several aspects of a comprehensive workplace health management seem to offer valuable information on how managing directors and leading employees of human resources departments in Germany think about such issues. Obviously, a non-controversial issue—and thus practically a ‘commonplace’—is that companies have a due social responsibility expressed by classical occupational health and safety obligations. Other attitudes, however, seem to be at least controversial if not overwhelmingly opposed; the latter is true when it comes to the attitude ‘positive view of *general* health services in the workplace’. Furthermore, a general skepticism toward workplace health management (cf. the third attitude in Table 5) seems to be widespread but not dominant. On the other hand, the attitude ‘workplace health management and workplace health promotion create added value to companies’ seems to be shared by surprisingly many company representatives. This latter result could be a ‘hook’ for systematic attempts to convince more companies that a structured workplace health management brings a benefit also to small and medium-sized companies. Against this background it might be useful in future campaigns to highlight paradigmatic ‘success stories’ of small and medium-sized enterprises which have clearly benefitted from establishing smart workplace health management components; such a strategy has been proposed already a decade ago [33]. A reasonable supplement of this strategy could—and should—be how to prevent typical mistakes and shortcomings [77]. In such campaigns, one should primarily focus on easy-to-implement ‘basic’ measures (such as occupational health and safety measures [33,77]) and leave aside the idea that *general* health services might also be implemented in companies—the latter idea is likely to seem too ‘out of touch’ to most company representatives.

Future research on the topic could, apart from verifying (or supplementing) the detected attitudes in independent samples, examine the relationship between company managers’ attitudes and the degree of implementation of workplace health management measures in companies. Furthermore, sociopolitical stakeholders could use the results of this study (and of subsequent studies) to better identify difficulties for—or limits to—future attempts to disseminate comprehensive workplace health management in companies. Moreover, the current COVID-19 pandemic may change the attitudes of company representatives towards health-related topics, too, as the pandemic goes along with new health-related measures in German companies (such as providing the employees with rapid SARS-CoV-2 antigen testing and offering COVID-19 vaccination in the company setting). Thus, it would be useful to investigate, using the attitude approach presented here, whether and to what extent the anti-COVID-19 measures in the companies have changed the attitudes of company representatives toward workplace health management.

## Figures and Tables

**Table 1:** Company characteristics according to company size (source: [4]).

Company Size	10–50 Employees	51–100 Employees	101–200 Employees	201–500 Employees	>500 Employees
Number of companies addressed	n = 570	n = 159	n = 89	n = 62	n = 26
Response (%/n)	22.3% n = 127	25.8% n = 41	31.5% n = 28	32.3% n = 20	23.1% n = 6
Occupational health physician available (%/n) *	29.1% n = 37	63.4% n = 26	78.6% n = 22	85.0% n = 17	83.3% n = 5
Occupational safety engineer available (%/n) ^‡^	63.0% n = 80	85.4% n = 35	100.0% n = 28	100.0% n = 20	100.0% n = 6

* According to regulation [72], an occupational health physician has to be available in all enterprises with more than 50 employees (in some branches, this limit is lower) and in the smaller enterprises in the case that the employer feels the need for occupational health counselling (so called “alternative, demand-based supervision”). ^‡^ An occupational safety engineer has to be available in all enterprises with more than 50 employees (in some branches, this limit is lower) and in the smaller enterprises in case the employer feels the need for occupational health counselling (so called “alternative, demand-based supervision”). In small enterprises (max. 50 employees), the employer can receive special training with regard to occupational health and safety by statutory accident insurance in order to reduce the need for support by occupational safety engineers [72].

**Table 2:** Sociodemographic characteristics of respondents (source: [4]).

Characteristic	Characteristic Values	
Position of respondent	Managing director	52.7% (n = 117)
	Member of personnel department	34.7% (n = 77)
	Other	11.7% (n = 26)
	Missing	0.9% (n = 2)
Gender of respondent	Male	54.1% (n = 120)
	Female	45.0% (n = 100)
	Missing	0.9% (n = 2)
Age of respondent (in years)	Mean	50.3
	Median	52.0
	Standard deviation	10.6
	Min-Max	25–82

**Table 3:** Total variance explained by the six extracted factors (based on n = 222 questionnaires).

Factor	Initial Eigenvalues of Factors and Explained Variance			Rotated Sum of Squared Factor Loads and Explained Variance (after 9 Iterations) ^+^		
	Total Eigenvalue *	Explained Variance (%)	Cumulated Explained Variance (%)	Total Eigenvalue	Explained Variance (%)	Cumulated Explained Variance (%)
1	7.7	29.5	29.5	3.9	14.9	14.9
2	2.3	8.7	38.2	3.1	12.1	27.0
3	2.1	8.0	46.2	2.7	10.3	37.3
4	1.5	5.0	51.8	2.4	9.4	46.7
5	1.4	5.4	57.2	1.9	7.4	54.0
6	1.1	4.1	61.4	1.9	7.3	61.4

* Factors were extracted according to the Kaiser criterion (initial total eigenvalue >1). ^+^ Rotation method: varimax.

**Table 4:** Assignment of individual items to underlying factors (attitudes).

Factor No. & Name	Item No.	Wording of the Declarative Statement (Item)	Factor Load ^#^
1. Positive view on *general* health services in the company	1.1	Offers for early disease detection (e.g., risk factors for heart attack) should not be made in the company.	−0.86
	1.2	Cancer screening services (e.g., colorectal cancer) should not be offered in the company.	−0.85
	1.3	Screening examinations for diseases that usually do not occur until retirement age should not be offered in the enterprise.	−0.78
	1.4	Early detection examinations for diseases that often occur before retirement age should be offered in the company.	0.64
	1.5	All employees should be offered vaccinations against non-work-related common diseases (e.g., tetanus) in the company.	0.55
	1.6	All employees should be offered vaccinations for private travel in the company.	0.51
2. WHM and WHP create added value for companies	2.1	Good implementation of workplace health management increases employees’ job satisfaction.	0.65
	2.2	Comprehensive, structured workplace health management is an investment that pays off.	0.65
	2.3	Workplace health promotion that is tailored to requirements helps to retain employees.	0.64
	2.4	Sustainable health promotion must strengthen employees’ personal responsibility for their own health.	0.59
	2.5	Comprehensive workplace health management can only work in larger companies.	−0.59
	2.6	Workplace health promotion that goes beyond legal requirements adds value to the company.	0.58
	2.7	It is sufficient for companies to fulfill the legal requirements for occupational health and safety and company reintegration management.	−0.43
3. General skepticism toward WHM	3.1	Workplace health management serves the image of the company rather than the health of the employees.	0.73
	3.2	Health is a private matter for each employee.	0.66
	3.3	Before society demands more commitment from companies, general health care should be improved.	0.58
	3.4	Workplace health management is difficult to implement in company practice.	0.49
4. Positive view on companies as setting for health promotion	4.1	Good workplace health promotion offerings can compensate for deficits in general health care (e.g., long waiting times).	0.69
	4.2	The company is particularly well positioned to strengthen employees’ personal responsibility for their own health.	0.66
	4.3	The workplace is the ideal place to address employees for health.	0.58
	4.4	Companies should not only address work-related health hazards, but also the general health of employees.	0.49
5. Positive view on … *	5.1	Companies contribute to maintaining the health of employees through good working conditions.	0.72
	5.2	Companies must avoid work-related health hazards to employees.	0.71
	5.3	Companies take on social responsibility through comprehensive workplace health management.	0.45
6. Positive view on… **	6.1	Anyone who has to measure blood pressure regularly should be able to do so at the company doctor’s practice.	0.83
	6.2	Chronically ill employees should also be able to have necessary laboratory check-ups performed by the company doctor.	0.74

Abbreviations: WHM—workplace health management; WHP—workplace health promotion. ^#^ Negative factor loadings result for those declarative statements that were formulated in a way that was opposite in content to the other statements of that factor. * 5. Positive view on occupational safety and health according to corporate social responsibility ** 6. Positive view on carrying out minor medical checks at the company doctor’s practice.

**Table 5:** Standardized agreement with six attitudes toward workplace health management (minimum agreement: 0, maximum agreement: 10).

Attitude	Mean of Agreement Score *	Standard Deviation	Range of Agreement Score	Count of Valid Agreement Score Values ** (n)
Positive view on *general* health services in the company (6 items)	3.26	2.23	0.00–10.00	198
Workplace health management and workplace health promotion create added value to companies (7 items)	6.08	1.80	0.00–10.00	179
General skepticism toward workplace health management (4 items)	5.02	1.99	0.00–10.00	196
Positive view on companies as setting for health promotion (4 items)	4.72	1.85	0.00–10.00	197
Positive view on occupational safety and health according to corporate social responsibility (3 items)	7.86	1.48	3.33–10.00	192
Positive view on carrying out minor medical checks at the company doctor’s practice (2 items)	4.91	2.71	0.00–10.00	168

* For the calculation of score values for a given attitude (‘factor’), response scales of items which had loaded negatively on ‘their’ factor, were reversed (cf. Section 2.3.2). This concerned items 1.1, 1.2, 1.3, 2.5, 2.7., and 3.1 (cf. Table 4). ** A valid agreement score was computed only for those respondents who had given valid answers to at least three quarters of the items forming a score (cf. Section 2.3.2 above).

## Data Availability

The data are not publicly available due to data use restrictions contained in study participants’ information material.
